# Elevated SNRPA1, as a Promising Predictor Reflecting Severe Clinical Outcome *via* Effecting Tumor Immunity for ccRCC, Is Related to Cell Invasion, Metastasis, and Sunitinib Sensitivity

**DOI:** 10.3389/fimmu.2022.842069

**Published:** 2022-02-23

**Authors:** Aimin Jiang, Jialin Meng, Wenliang Gong, Zhonghua Zhang, Xinxin Gan, Jie Wang, Zhenjie Wu, Bing Liu, Le Qu, Linhui Wang

**Affiliations:** ^1^ Department of Urology, Changhai Hospital, Naval Medical University (Second Military Medical University), Shanghai, China; ^2^ Department of Urology, The First Affiliated Hospital of Anhui Medical University; Institute of Urology, Anhui Medical University; Anhui Province Key Laboratory of Genitourinary Diseases, Anhui Medical University, Hefei, China; ^3^ Department of Clinical Pharmacy, No. 988 Hospital of Joint Logistic Support Force, Zhengzhou, China; ^4^ Department of Urology, The Third Affiliated Hospital, Naval Medical University (Second Military Medical University), Shanghai, China; ^5^ Department of Urology, Affiliated Jinling Hospital, Medical School of Nanjing University, Nanjing, China

**Keywords:** clear cell renal cell carcinoma, small nuclear ribonucleoprotein polypeptide A, tumor immunity, prognosis, drug response

## Abstract

Clear cell renal cell carcinoma (ccRCC) is the most common subtype of renal carcinoma and is associated with poor prognosis and notorious for its immune dysfunction characteristic. SNRPA1 is a spliceosome component responsible for processing pre-mRNA into mRNA, while the biological effect of SNRPA1 in ccRCC remains elusive. The aim of this study was to decipher the effect of SNRPA1 on clinical effect and tumor immunity for ccRCC patients. Multi-databases were collected to evaluate the different expression, prognostic value, DNA methylation, tumor immune microenvironment, and drug sensitivity of SNRPA1 on ccRCC. IHC was utilized to validate the expression and prognostic value of SNRPA1 in ccRCC patients from the SMMU cohort. The knockout expression of SNRPA by sgRNA plasmid inhibited the cell proliferation, migration, and metastasis ability and significantly increased the sensitivity of sunitinib treatment. In addition, we explored the role of SNRPA1 in pan-cancer level. The results indicated that SNRPA1 was differentially expressed in most cancer types. SNRPA1 may significantly influence the prognosis of multiple cancer types, especially in ccRCC patients. Notably, SNRPA1 was significantly correlated with immune cell infiltration and immune checkpoint inhibitory genes. In addition, the aggressive and immune inhibitory effects shown in SNRPA1 overexpression and the effect of SNRPA1 on ccRCC cell line invasion, metastasis, and drug sensitivity *in vitro* were observed. Moreover, SNRPA1 was related to Myc, MTORC, G2M, E2F, and DNA repair pathways in various cancer types. In all, SNRPA1 may prove to be a new biomarker for prognostic prediction, effect tumor immunity, and drug susceptibility in ccRCC.

## Introduction

Worldwide, about 350,000 individuals were diagnosed with renal cell carcinoma (RCC) per annum, which is the most frequent type of malignant tumor of the kidney (1). Until now, obesity, hypertension, tobacco, and NSAID use were proved to be the risk factors for the development of RCC (2). To be specific, clear cell RCC (ccRCC) (75%), papillary RCC (16%), and chromophobe RCC (7%) took the majority of the histological subtypes of RCC (3). ccRCC is characterized by inactivation of the von Hippel-Lindau tumor suppressor gene (VHL), which led to the abnormal accumulation of HIF proteins, regulating angiogenesis, glycolysis, and apoptosis (2). Further genomic research on ccRCC has discovered more common mutations including BAP-1, PBRM1, SETD2, and PIK3CA (4). Notwithstanding the development of multiple targeted agents in the past few years, surgery remains the first option for the patients with early and local ccRCC (1, 5). However, late-stage ccRCC has a poor prognosis, accompanied by a 5-year survival of less than 10% and limited treatment methods (6). Thus, there is an urgent demand for reliable prognostic markers and therapeutic targets.

In eukaryotes, non-coding introns are removed from precursor messenger RNAs (pre-mRNAs) by ribonucleoproteins (RNPs) called spliceosome; meanwhile, exons are linked to form protein-coding mRNAs (7). The spliceosome consists of small nuclear RNAs (snRNAs) and five small nuclear ribonucleoproteins (snRNPs) (U1, U2, U4, U5, and U6 snRNPs) (8). Dysfunction of assembly or splicing reactions may lead to disease pathogenesis. As a crucial component of U2 snRNP, small nuclear ribonucleoprotein polypeptide A (SNRPA1) is a potential biomarker for the prognosis of various cancers (9, 10).

SNRPA1 has been reported to be upregulated in cancers (11, 12). Fish et al. (13) revealed that SNRPA1 can interact with gene enhancers to promote the transcription of cassette exon and results in the metastatic colonization and cell invasion of lung cancer, through the SNRPA1-mediated regulation of PLEC alternative splicing. In colorectal cancer (CRC) cells, SNRPA1 binds directly to the BCL-2-associated athanogene-1 (BAG-1) mRNA to decrease the expression of its outcome. BAG-1 is considered to be a symbol of poorer prognosis, which means the presence of SNRPA could be beneficial to CRC patients (14). On the contrary, SNRPA1 has been found to promote CRC formation through the upregulation of NRP1 and PIK3R1 and downregulation of E2FZ, VEGFC, MKI67, and CDK1 (15). Dou et al. found out that the gastric cancer (GC) patients with a higher expression of SNRPA have an obvious shorter overall survival time than the opposite (12). They discovered that the nerve growth factor (NGF) was upregulated with SNRPA overexpression at the mRNA and protein levels, suggesting that NGF is probably a downstream effect in GC. Yuan et al. (16) found that the SNRPA1 expression level was positively associated with Gleason score in prostate cancer (PCa). Furthermore, inhibition of SNRPA1 could result in a decrease in PCa cell migration, proliferation, and colony formation. To sum up, the role of SNRPA1 has not been fully understood.

Whether SNRPA1 plays a critical role in the ccRCC has yet to be clarified. In this study, SNRPA1 was found to be upregulated in ccRCC tissues and was correlated with the migration and invasion of tumor cells. Therefore, the aim of the study was to illustrate the function and mechanistic basis of SNRPA1 in ccRCC. Besides, we investigated the predicted function of SNRPA1 assisting in determining clinic pathological features and prognosis in ccRCC patients.

## Methods and Materials

### Data Collection and Preprocessing

Normalized expression profile data, TMB data, MSI data, and clinical information of pan-cancer including clear cell renal carcinoma were download from the UCSC Xena database (17, 18). Oncomine (https://www.oncomine), a web-based data mining platform assembling 86,733 samples and 715 gene expression datasets, was utilized to validate the expression difference of SNRPA1 among pan-cancer (19). As for the out-house datasets, GSE29609 (containing 39 ccRCC samples) and EGAS00001000509 (containing 100 ccRCC samples) were used to validate the prognostic value of SNRPA1. For datasets in the UCSC Xena and Oncomine databases, institutional review board approval and informed consent were not required. Patients were excluded if they 1) did not have prognostic information and 2) died within 30 days.

### Differential Expression Analysis and Validation of SNRPA1

Analysis of SNRPA1 expression among pan-cancer was performed in the Oncomine database, using p value <0.05 and absolute fold change >1.5 as the threshold. The same threshold was implied to identify the different expression levels of SNRPA1 in the TCGA database. p-value cutoff = 0.01, log2 fold change (FC) cutoff = 1, and “Match TCGA normal and GTEx data” were set as criteria. The log2 (transcripts per million (TPM) +1) transformed expression data were applied for the box or violin plots.

### Enrichment Analysis of SNRPA1

Based on the guilt of association of single genes in the expression profile, Pearson’s correlation between SNRPA1 and other mRNA retrieved from the TCGA transcriptome data were analyzed. Sorted by the level of association index between genes and SNRPA1, those genes most related to SNRPA1 expression were selected for enrichment analysis (the threshold of most correlated genes is absolute correlation coefficient >0.3 and p value <0.05). R package “clusterprofiler” was used to perform Gene Ontology (GO) analysis, Kyoto Encyclopedia of Genes and Genomes (KEGG) analysis, and Gene Set Enrichment Analysis (GSEA) (20) based on the most related genes selected which were mentioned above.

### Assessment of Potential Chemotherapy Drugs to SNRPA1 Expression

Clinical characteristics including tumor stage and drug sensitivity were introduced, and the relationship between SNRPA1 expression and those characteristics was analyzed. The data including IC50 (half-maximal inhibitory concentration) and gene expression of cancer cell lines were downloaded from the CellMiner database (https://discover.nci.nih.gov/cellminer/home.do) and GDSC (https://www.cancerrxgene.org/) database, respectively (21, 22).

### Differences in Tumor Microenvironment and Immunotherapy Response

R package “ESTIMATE” was introduced to evaluate the relationship between infiltration degree of immune and stromal cell and expression of SNRPA1 in pan-cancer. A co-expression analysis of immune-related gene and SNRPA1 was performed *via* R package “ggpubr” and “ggcor.” R package “CIBERSORT” was used to quantify the immune cell infiltration scores among pan-cancer, then the correlation of degree of immune cell and SNRPA1 expression was calculated. In addition, the correlation between neoantigen count, TMB, MSI, and expression of T cell exhaustion marker genes (including PDCD1, TIGIT, CD274, CTLA4, LAG3, CXCL13, LAYN, and HAVCR2), DNA mismatch repair system genes (including MLH1, MSH2, MSH6, PMS2, and EPCAM), DNA methyltransferase (including DNMT1, DNMT2, DNMT3A, and DNMT3), and ESTIMATE scores and SNRPA1 expression was analyzed. We also calculated the immune infiltration scores *via* the ssGSEA algorithm and analyzed the correlation and difference between immune cell infiltration and SNRPA1 expression level in ccRCC. The TIMER website (http://timer.cistrome.org/) was utilized to validate the influence of SNRPA1 mutation on immune cell infiltration in ccRCC (23).

### Validation Different Expression of SNRPA1

RT-qPCR, Western blotting, and immunohistochemical (IHC) staining were performed to validate SNRPA1 expression in paired tumor and adjacent renal tissue (including 40 clear cell renal carcinoma tissues from Changzheng Hospital). Primer sequences for RT-qPCR were as follows: primer for SNRPA1 (forward primer: GGTGCTACGTTAGACCAGTTTG, reverse primer: GTCCCTCACCTATACGGCATATT) and primer for GAPDH (forward primer: GGAGCGAGATCCCTCCAAAAT, reverse primer: GGCTGTTGTCATACTTCTCATGG). Antibodies including SNRPA1 (SNRPA1 polyclonal antibody, EPR7557, Abcam, Cambridge, MA, USA) and YY1 (YY1 polyclonal antibody, EPR4652, Abcam) were purchased from Abcam Trading Co., Ltd. The detailed procedure referred to our previous researcher’s protocols. All those results were analyzed by R software.

### Investigation of SNRP1 Biological Function *In Vitro*

Human normal renal epithelial cell line HK-2 and cancer cell lines (including 769-P, 786-O, A-498, Caki-1, Caki-2, and OS-RC-2) were obtained from the American Type Culture Collection (ATCC). Cell lines were cultured according to the instructions. The Cas9/gRNA of SNRPA1 was chemically synthesized by Shanghai GeneChem Co., Ltd. Renal cell lines (including 786-O and OS-RC-2) were transduced with Cas9 lentiviral particles and selected with puromycin for 10 days. The detailed procedures were referred to operation manual provided by Shanghai GeneChem Co., Ltd. QT-PCR and Western blotting were applied to verify the knockout efficiency of the Cas9 virus. CCK-8 (Cell Counting Kit-8) was used to detect the cell viability between negative control and SNRPA1-knockout groups. Scratch assay experiment and transwell migration assay were used to evaluate cell migration ability. A colony-forming experiment was conducted to determine the ability of reproduction *in vitro*. The detailed procedure of *in vitro* experiments is referred to our previous study. All experiments above were carried out in three replications and repeated three times.

### Statistical Analysis

Differences in the expression of the SNRPA1 in the public data sets were compared by one-way ANOVA, and differences in clinical information and immune checkpoint inhibitor response between the two different subgroups were compared by the chi-squared test. Differences in OS and PFI between the two subgroups were compared by the Kaplan–Meier method and log-rank rest. The hazard ratios (HRs) were calculated by univariate Cox regression and multiple Cox regression analysis. All the image analysis of this study was performed using ImageJ software. All p-values were two-sided, with p < 0.05 as statistically significant. The adjusted p-value was obtained by Benjamini–Hochberg (BH) multiple-test correction. All data processing, statistical analysis, and plotting were conducted using R 4.0.4 software.

## Results

### SNRPA1 Expression Elevated in Tumor Tissues as Compared With Normal Tissues

**Figure 1:**
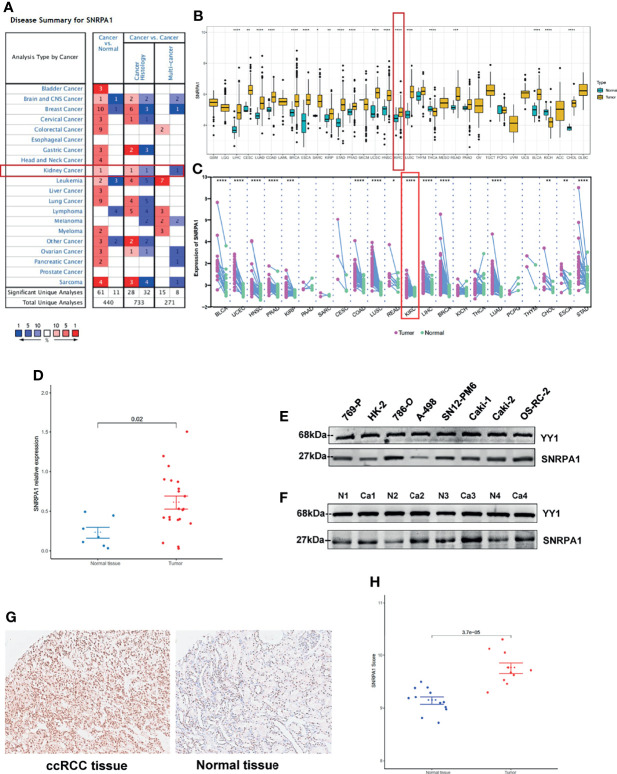
Elevated expression pattern of SNRPA1 in ccRCC. **(A)** Expression of SNRPA1 in 20 different cancer types from the Oncomine database. **(B)** Differential expression of SNRPA1 in 33 cancer types from the TCGA database. **(C)** Differential expression of SNRPA1 in paired cancer and normal tissues of 22 cancer types from the TCGA database, *p < 0.05, **p < 0.01, ***p < 0.001, ****p < 0.0001. **(D)** RT-PCR result of SNRPA1 expression in renal cancer and adjacent normal tissue from Changzheng Hospital, n = 40, p < 0.01. **(E)** Western blotting of SNRPA1 differential expression in kidney normal and tumor cell lines. **(F)** Western blotting of SNRPA1 differential expression in tumor tissue and adjacent normal tissue from ccRCC patients. **(G)** Representative immunohistochemical images of SNRPA1 expression in ccRCC and adjacent tissues from Changzheng Hospital, scale bar = 50 μm. **(H)** Quantification of the H-score for SNRPA1 protein level assessed by immunohistochemical assay.

We firstly used the Oncomine online database to reveal the diverse expression patterns of SNRPA1 among tumor and normal tissues in pan-cancer and revealed the elevated level of SNRPA1 in tumor tissues (**Figure 1A**); further validation of the increased expression of SNRPA1 was performed with the pan-cancer data from the TCGA project with (**Figure 1B**) and in cancer vs. paired adjacent tissues (**Figure 1C**). Interestingly, we observed the high level of SNRPA1 in ccRCC samples as compared with normal samples (all p < 0.005, **Figures 1B, C**). As for the human samples, we collected the fresh tissues of ccRCC and adjacent normal renal tissues and validated the SNRPA1 in mRNA and protein levels; SNRPA1 mRNA and protein levels are significantly increased in tumor tissues as compared with adjacent normal tissues (p < 0.001, **Figures 1D, E**). We further confirmed the elevated SNRPA1 protein level in ccRCC in cell lines and human samples. As compared to normal renal cell line HK2, we observed the increased level of SNRPA1 in ccRCC cell lines, especially for 786-O and OS-RC-2 cell lines (**Figure 1F**). The FFPE slides from ccRCC patients were also employed to verify the elevated SNRPA1 protein level in ccRCC by IHC staining; we observed that tumor slides contained the higher level of SNRPA1 H-score (p <0.01, **Figures 1G, H**). Moreover, the clinical features of the patients are summarized in **Supplementary Table 1**. To assess whether SNRPA1 was expressed at different levels in various cancer stages, different pathological stages (I, II, III, and IV) of pan-cancer were collected. The results showed that the expression level of SNRPA1 was significantly different in different stages of various cancer types (**Supplementary Figure 1**), suggesting that SNRPA1 may play an important role in progression of various carcinomas.

### Correlation of SNRPA1 Expression With DNA Methylation and RNA Modification

**Figure 2:**
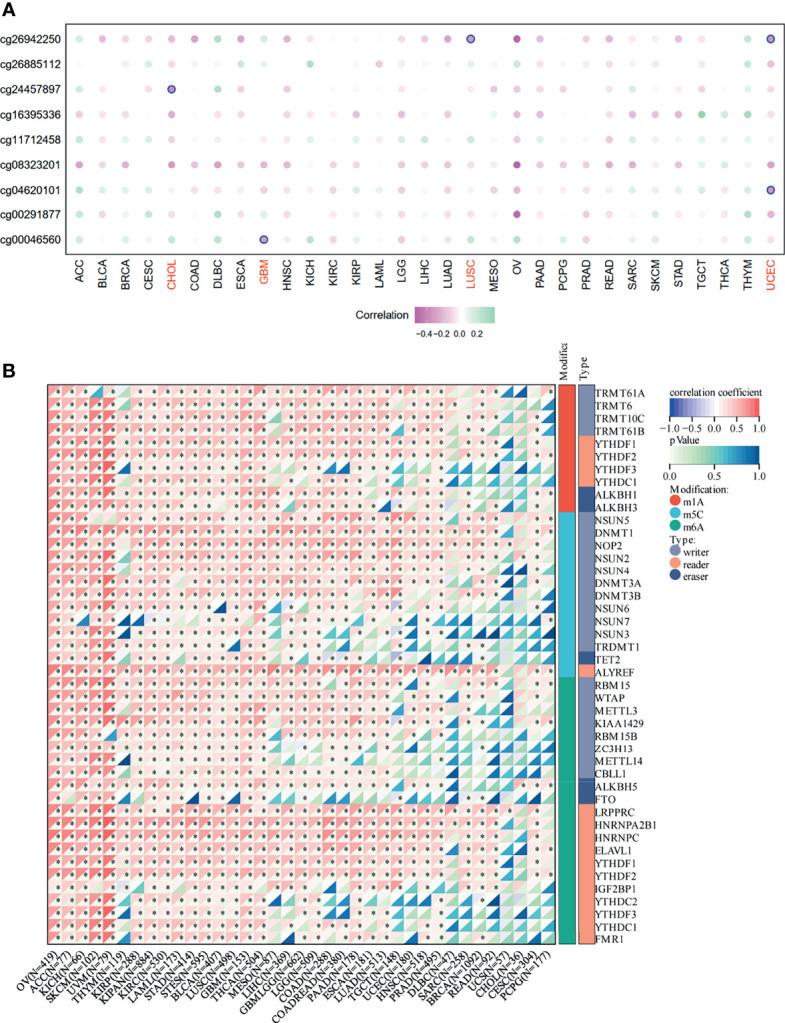
DNA methylation and RNA modification in SNRPA1. **(A)** The correlation of SNRPA1 expression and methylation degree in pan-cancer. **(B)** The correlation of SNRPA1 expression and RNA modification regulator expression in pan-cancer. *p < 0.05.

To further analyze the potential regulation effect of DNA methylation and RNA modification in SNRPA1 expression, firstly, we systematically explored the correlation of DNA methylation level and SNRPA1 expression, which indicated that DNA methylation could negatively regulate SNRPA1 expression in CHOL *via* cg24457897, GBM *via* cg00046560, LUSC *via* cg26942250, and UCES *via* cg0620101 (**Figure 2A**); RNA modification-related genes (including m1A, m5C, and m6A) were also significantly positively correlated with SNRPA1 expression (**Figure 2B**). All those results indicated that SNRPA1 expression might mainly regulated *via* RNA posttranscriptional modification.

### Elevated SNRPA1 Level Indicated Severe Clinical Outcome for ccRCC Patients

**Figure 3:**
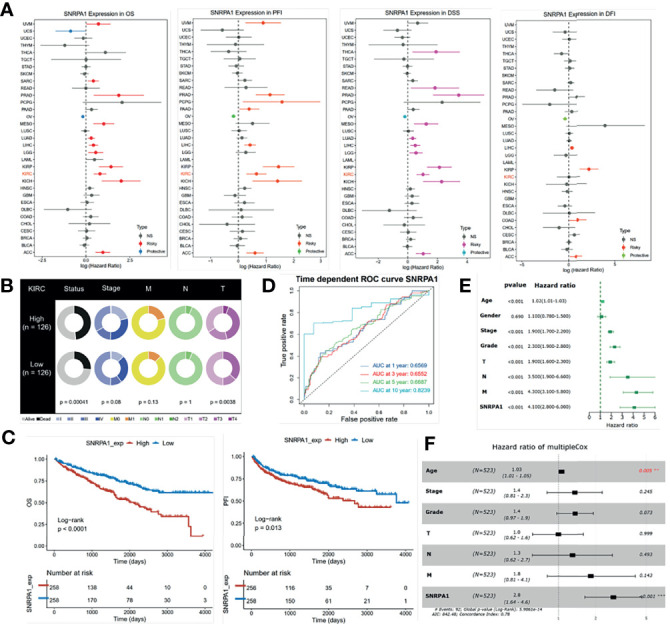
Prognostic value of SNRPA1 in ccRCC. **(A)** Relationship between SNRPA1 expression level and prognosis (from left to right is OS, PFI, DSS, and DFI) of pan-cancer from the TCGA database. **(B)** Different distribution of clinical features of ccRCC patients with high expression and low expression of SNRPA1. **(C)** Survival curves of OS and PFI in ccRCC patients from the TCGA database. **(D)** Time-dependent ROC curve of OS based on SNRPA1 expression. **(E, F)** Univariate and multivariate Cox regression of SNRPA1 expression and other clinical characteristics.

We first assessed the prognostic value of SNRPA1 in pan-cancer of OS, PFI, DSS, and DF1 (**Figure 3A**). SNRPA1 acted as the risk factor to OS in 12 tumor types, to PFI in 9 tumor types, to DSS in 11 tumor types, and to DFI in 4 types (**Figure 3A**). The clinical characteristics of SNRPA1-high and -low expressed subgroups of ccRCC are summarized in **Table 1**. Interestingly, SNRPA1 is the risk factor to all four types of prognosis in TCGA-KIRC ccRCC patients. Further analysis of the SNRPA1 association to clinical features revealed that patients with a high level of SNRPA1 met more death and mostly in advanced tumor stages (**Figure 3B**). The 5-year OS rate of SNRPA1-high patients is only 53.67%, while 73.79% SNRPA1-low patients alive at the end observe a 5-year point (p < 0.001, **Figure 3C** upper); the diverse outcome of PFI is similar (p = 0.016, SNRPA1-high: 59.45% vs. SNRPA1-low: 69.84%, **Figure 2C** lower). In addition, the prognostic value of SNRPA1 was further confirmed in GSE29609 (p = 0.014) and EGAS00001000509 (p = 0.032) cohorts; a higher expression of SNRPA1 predicted the unfavorable prognosis in these two cohorts as well (**Supplementary Figure 2**). The prognostic value of SNRPA1 assessed by the ROC curve indicated the 1-year AUC value of 0.6569, 3-year AUC value of 0.6552, 5-year AUC value of 0.6687, and 10-year AUC value of 0.8239 (**Figure 3D**). SNRPA1 level, patient age, tumor stage, and tumor grade were all the prognostic factors for ccRCC patients (**Figure 3E**), and SNRPA1 still acted as the independent prognostic risk factor after adjusting for clinical features (p < 0.001, **Figure 3F**).

**Table 1:** Clinical characteristics of SNRPA1 -high and -low expressed ccRCC patients.

	SNRPA1 High	SNRPA1 Low	p-value
	(N = 258)	(N = 259)	
Gender			
FEMALE	99 (38.4%)	81 (31.3%)	0.109
MALE	159 (61.6%)	178 (68.7%)	
Age (years)			
Age <40	9 (3.5%)	8 (3.1%)	0.994
Age ≥ 40	249 (96.5%)	251 (96.9%)	
Race			
ASIAN	6 (2.3%)	2 (0.8%)	0.0493
BLACK OR AFRICAN AMERICAN	32 (12.4%)	19 (7.3%)	
WHITE	220 (85.3%)	238 (91.9%)	
Laterality			
Bilateral	1 (0.4%)	0 (0%)	0.596
Left	120 (46.5%)	119 (45.9%)	
Right	137 (53.1%)	140 (54.1%)	
T			
T1	114 (44.2%)	154 (59.5%)	0.00177
T2	40 (15.5%)	27 (10.4%)	
T3	95 (36.8%)	76 (29.3%)	
T4	9 (3.5%)	2 (0.8%)	
N			
N0	118 (45.7%)	116 (44.8%)	0.687
N1	9 (3.5%)	6 (2.3%)	
NX	131 (50.8%)	137 (52.9%)	
M			
M0	188 (72.9%)	226 (87.3%)	<0.001
M1	45 (17.4%)	30 (11.6%)	
MX	25 (9.7%)	3 (1.2%)	
Stage			
Stage I	110 (42.6%)	152 (58.7%)	0.00306
Stage II	32 (12.4%)	23 (8.9%)	
Stage III	67 (26.0%)	53 (20.5%)	
Stage IV	49 (19.0%)	31 (12.0%)	
Grade			
G1	8 (3.1%)	6 (2.3%)	0.127
G2	105 (40.7%)	118 (45.6%)	
G3	105 (40.7%)	98 (37.8%)	
G4	40 (15.5%)	32 (12.4%)	
GX	0 (0%)	5 (1.9%)	
Event			
Yes	107 (41.5%)	62 (23.9%)	<0.001
No	151 (58.5%)	197 (76.1%)	
Event_PFI			
Yes	87 (33.7%)	68 (26.3%)	0.079
No	171 (66.3%)	191 (73.7%)	

### Identify SNRPA1 Mostly Impacted Signaling Pathways

**Figure 4:**
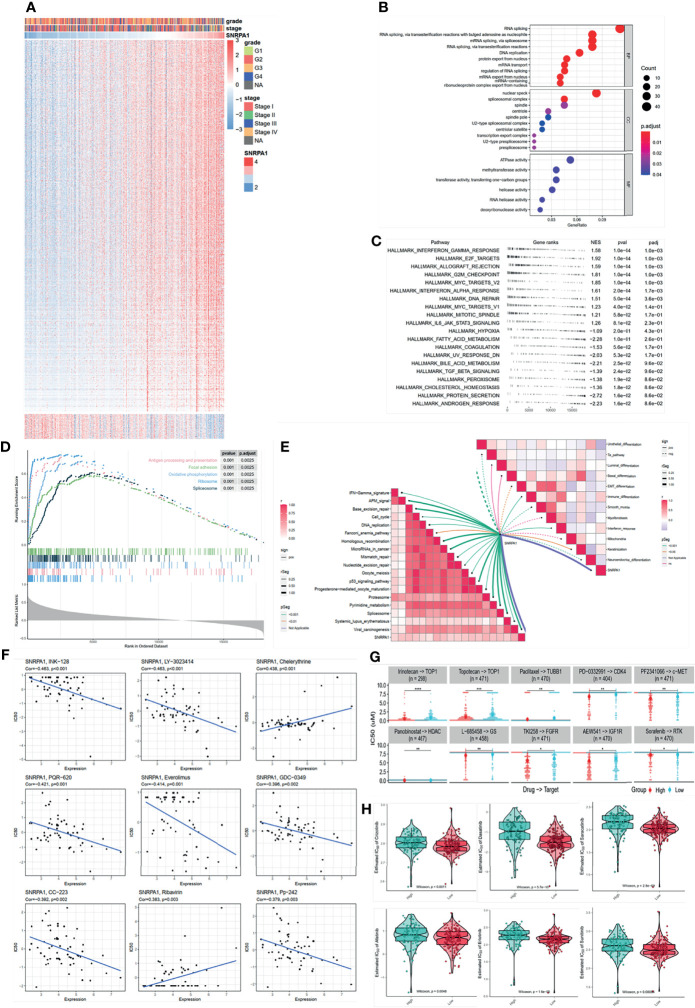
SNRPA1 impacted signaling pathways and potential effective chemotherapy drugs. **(A)** Heatmap of SNRPA1 co-expressed genes in ccRCC patients. **(B–D)** GO, KEGG, and GSEA analyses of SNRPA1 in ccRCC. **(E)** Correlation of SNRPA1 expression level and urinary system carcinoma-related signature. **(F)** Correlation of SNRPA1 expression level and IC50 of different drugs obtained from the CellMiner database. **(G)** Different IC50 levels of drugs between SNRPA1 high- and low-expression groups obtained from the CCLE database. **(H)** Different IC50 levels of target drugs between SNRPA1 high- and low-expression groups based on the GDSC database.

To in-depth dig out SNRPA1 involved in the biological process, we first collected a total of 500 genes with the correlation higher than 0.5 to SNRPA1 (**Figure 4A**). GO terms pathway enrichment analysis revealed that SNRPA1 impacted the activation of RNA splicing, DNA replication, mRNA transport, nuclear speck, ATPase activity, and RNA helicase activity (**Figure 4B**). Furthermore, we calculated the activation status of HALLMARKER tumor signatures between SNRPA1 high and low groups, revealing that high-level SNRPA1 correlated with the activation of G2M checkpoint, MYC targets, E2F targets, interferon gamma response, IL6/JAK/STAT3 signaling, and interferon alpha response pathways, while low SNRPA1 correlated with the activation of androgen response, protein secretion, fatty acid metabolism, and hypoxia pathways (**Figure 4C**). GSEA analysis presented the similar results; high SNRPA1 activated the signaling pathway of antigen processing and presentation, focal adhesion, and oxidative phosphorylation (**Figure 4D**). We also compared the association between SNRPA1 and therapeutic signatures, including targeted therapy-associated gene signature, gene signatures predicting radiotherapy, and signatures predicting response of immunotherapy. We observed that SNRPA1 positively associated with the signature of interferon response, immune differentiation, keratinization, cell cycle, DNA replication, mismatch repair, p53 signaling, and spliceosome and negatively associated with the signature of urothelial differentiation, Ta pathway, EMT differentiation, and neuroendocrine differentiation (**Figure 4E**).

### Potential Chemo Drugs for SNRPA1 Determined ccRCC Progress

A precise therapy for patients is needed in recent days; we tried to identify the potential chemo drugs to inhibit the SNRPA1-regulated oncogenic process. We revealed that SNRPA1 expression is negative with the IC50 of INK-128, LY-3023414, PQR-620, everolimus, GDC-0349, CC-223, and Pp-242, which means that these chemo drugs are suitable for the treatment with high level of SNRPA1, while chelerythrine and ribavirin might not be suitable (**Figure 4F**). Another database was also enrolled and presented that irinotecan, topotecan, paclitaxel, PD-0332991, PF2341066, panobinostat, L-685458, TKI258, AEW541, and sorafenib are suitable for patients with lower SNRPA1 (**Figure 4G**). Chemo drugs recorded in the GDSC database also revealed that patients with high SNRPA1 are more suitable for the treatment of crizotinib, dasatinib, saracatinib, afatinib, erlotinib, and sunitinib (**Figure 4H**).

### SNRPA1 Positively Associated With the Activated Immune Microenvironment of ccRCC Patients

**Figure 5:**
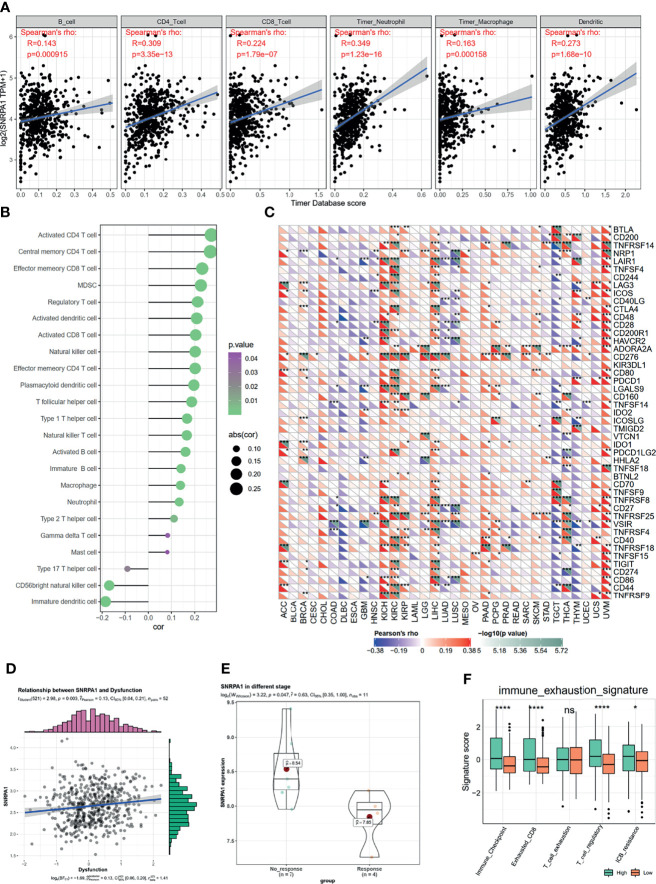
Correlation between SNRPA1 and ccRCC immune microenvironment, immunotherapy. **(A)** SNRPA1 is associated with immune cell infiltration in ccRCC obtained from the TIMER database. **(B)** Associations between SNRPA1 expression and infiltration scores of 28 immune cells in ccRCC patients. **(C)** Correlation between immune check points and SNRPA1 expression in pan-cancer. **(D)** Correlation between immune dysfunction score and SNRPA1 expression in ccRCC. **(E)** SNRPA1 expression in ccRCC patients’ response or no response to anti-PD-1 therapy. **(F)** Different enrichment scores of immune exhausted scores in SNRPA1 high and low ccRCC patients. *p < 0.05, **p < 0.01, ***p < 0.001, ****p value is too small and is close to zero. ns, p > 0.05.

The immune microenvironment plays a pivotal role in the genesis and progression of tumors. After analyzing the SNRPA1 most correlated genes and pathways as shown in **Figure 4**, we also observed several activated immune-associated signaling pathways. We further evaluated the correlation between SNRPA1 expression and immunocyte infiltration and observed that SNRPA1 significantly positively associated with the infiltration of B cells, CD4 T cells, CD8 T cells, neutrophil, macrophage, and dendritic cells (all p < 0.001, **Figure 5A**). Another calculation of immunocyte signatures by the ssGSEA algorithm also revealed the positive association between SNRPA1 and 20 types of immunocytes (**Figure 5B**). Immune checkpoints are important key components for the clinical immunotherapy for tumors; we evaluated the association between SNRPA1 and 47 immune checkpoints in pan-cancer. The results indicated that the SNRPA1 level was significantly associated with the increased level of immune checkpoints in ccRCC, especially for PDCD1 (PD-1), CD274 (PD-L1), PDCD1LG2 (PD-L2), and CTLA4 (**Figure 5C**). Although the increased level of SNRPA1 correlated with the infiltration of immunocytes and the elevated level of immune checkpoints, we also revealed that high SNRPA1 linked with the increased score of immune dysfunction and exclusion state which was assessed by the TIDE algorithm (r = 0.13, p = 0.003, **Figure 5D** and **Supplementary Figure 3**). Moreover, with the gene expression profile from an anti-PD-L1 treatment ccRCC cohort (24), we found that these patients who responded to the immunotherapy contained a lower level of SNRPA1 (**Figure 5E**). To clear up the inconsistency in immunocyte infiltration and response results to immunotherapy, we assessed the immune exhausted status in SNRPA1-high and -low level subgroups then revealed that although patients with high SNRPA1 contained the high proportion of immunocytes, they also met the immune exhaustion (**Figure 5F**); therefore, patients with low SNRPA1 might response more from anti-PD-L1 therapy.

### Knockdown of SNRPA1 Expression Reduced Cell Proliferation, Migration, and Invasion of ccRCC Cells

**Figure 6:**
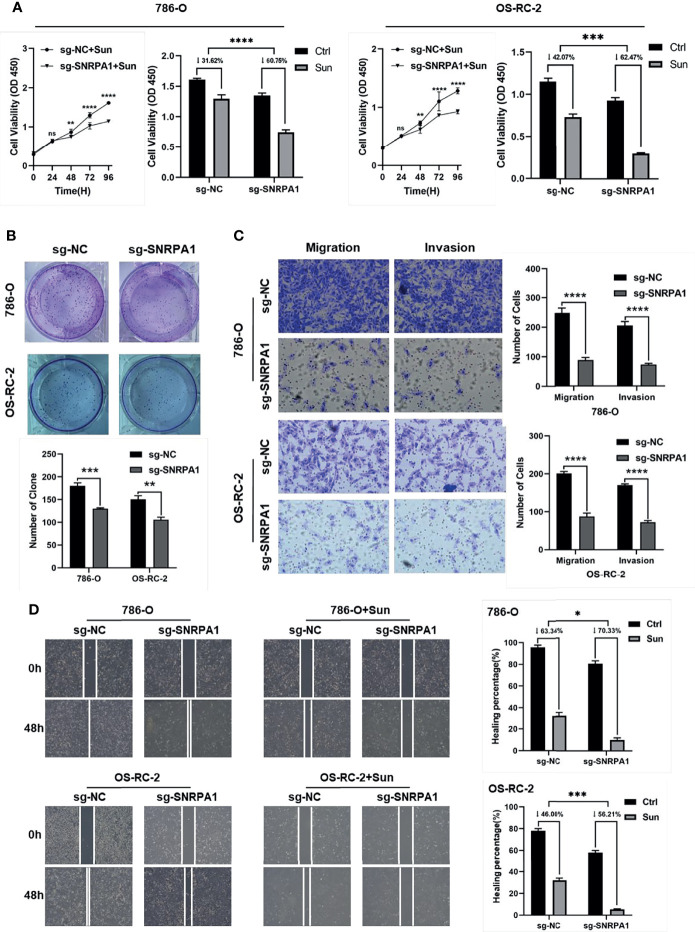
Validation the function of SNRPA1 with *in vitro* experiments by 786-O and OS-RC-2 cell lines. **(A)** Cell proliferation of 786-O and OS-RC-2 (right: treated with sunitinib) after being transfected with NC and SNRPA1 cas9-sgRNA plasmid, ***p < 0.001. **(B)** Clone formation ability of 786-O and OS-RC-2 after being transfected with NC and SNRPA1 cas9-sgRNA plasmid, ***p < 0.001. **(C)** Wound healing of 786-O and OS-RC-2 (right: treated with sunitinib) after being transfected with NC and SNRPA1 cas9-sgRNA plasmid, ***p < 0.001. **(D)** Cell migration and migration of 786-O and OS-RC-2 after being treated with NC and SNRPA1 cas9-sgRNA plasmid, ***p < 0.001, scale bar = 200 μm. *p < 0.05, ****p value is too small and is close to zero.

Firstly, we knocked down the SNRPA1 protein level by sg-SNRPA1 in both 786-O and OS-RC-2 cell lines, which obtained the higher level of SNRPA1 as compared to control HK-2 renal cells and confirmed the knockdown by WB (**Supplementary Figure 4A**). The cell viability decreased significantly from the 48- to 96-h results in sg-SNRPA1 groups as compared with sg-NC groups in both 786-O and OS-RC-2 cell lines (**Supplementary Figure 4B**). In addition, we revealed that patients with lower SNRPA1 was more sensitive to sunitinib treatment, as shown in the abovementioned results (**Figure 6A**); therefore, we validated the alterations of cell viability with the treatment of sunitinib, and we also observed the significant inhabitation by the knockdown of SNRPA1 to the cell viability of 786-O and OS-RC-2 cell lines (**Figure 6A**). Colony formation assay was also performed to compare the cell proliferation; we observed the results that knockdown of SNRPA1 can decrease the number of colonies in both 786-O and OS-RC-2 cell lines (**Figure 6B**), as well as the function of cell migration and cell invasion (**Figure 6C**). Furthermore, we also evaluated the impaction of cell migration by wound healing assay in sg-NC and sn-SNRPA1 subgroups treated with or without sunitinib. We observed that knockdown of SNRPA1 can decrease about 20%–30% cell migration, and this rate rose to more than 50% in sunitinib-treated groups (**Figure 6D**); therefore, the target therapy to SNRPA1 might be a potential new strategy for sunitinib resistance patients.

### Overview of SNRPA1 in Pan-Cancer

**Figure 7:**
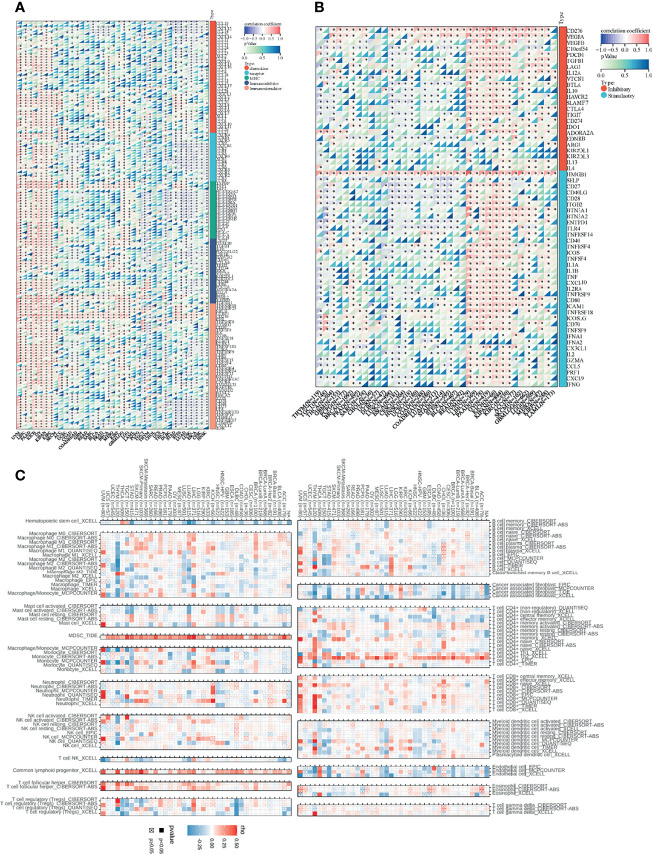
Association between SNRPA1 and tumor immunity in pan-cancer. **(A, B)** Correlations of SNRPA1 expression with immune moderator genes and immune checkpoint-related genes in 33 cancer types. **(C)** Correlation between SNRPA1 expression and immune cell infiltration *via* multiple algorithms.

Since there are few studies of SNRPA1 in cancers, especially in tumor immunity, herein we performed a comprehensive analysis of correlation analysis of SNRPA1 expression and immune-regulated genes, immune checkpoint inhibitor genes, and immune cell infiltration degree in pan-cancer levels. As **Figure 7A** shows, SNRPA1 expression was significantly positively correlated with chemokine, chemokine receptors, MHC, and immuno-inhibitor and immuno-stimulator genes in multiple cancer types including UVM, LIHC, PAAD, KICH, OV, KIPAN, and KIRC, but negatively related to those genes in TCGT, THYM, DLBC, STAD, STES, LUAD, LUSC, ESCA, CESC, and HNSC (**Figure 7A**). In addition, SNRPA1 expression was positively correlated with immune inhibitory and stimulatory genes in several cancer types UVM, LIHC, PAAD, KICH, OV, KIPAN, and KIRC, but negatively related in THYM, TGCT, THCA, LUSC, ESCA, CESC, and STES (**Figure 7B**). Concurrently, we analyzed the effect of SNRPA1 expression on immune cell infiltration in several algorithms including CIBERSORT, XCELL, EPIC, QUANTISEQ, and TIDE. As it is shown in **Figure 7C**, SNRPA1 was significantly negatively correlated with hematopoietic stem cells, cancer-associated fibroblasts, and endothelial cells in most cancer types, but positively related with MDSC, common lymphoid progenitor cells, and CD4 Th2 cells in various cancer types. Interestingly, SNRPA1 could regulate the infiltration of most immune cell types in BRCA, LIHC, LUAD, THCA, and THYM, which indicated that SNRPA1 could be treated as a promising target to regulate tumor immunity in those cancer types (**Figure 7C**). To explore the biological function of SNRPA1 in different cancer types, we firstly utilized the GSVA to calculate the enrichment scores of 50 canonical tumor associated pathways in pan-cancer level; then the correlation of those enrichment scores and SNRPA1 expression was estimated. The results indicated that SNRPA1 mainly positively regulated Myc, MTORC1, G2M checkpoint, E2F target, and DNA repair in most cancer types (**Supplementary Figure 5**). All those results indicated that SNRPA1 could regulate immune cell infiltration *via* different mechanisms under various tumor microenvironments, and further experiment needs to decipher the heterogeneous mechanisms.

## Discussion

ccRCC, as the most prevalent type of renal cell carcinoma, increased the health burden worldwide (5). In China, the incidence of RCC continuously increased from 1988 to 2014, which might be caused by the elevated number of aging populations, popularization of early health screening, and alteration of lifestyle; the incidence of RCC rose to 4.99/105 in 2014 (25). Therefore, it is necessary to identify the new strategy to enhance the precise diagnostic and clinical treatment. As a crucial component of U2 snRNP, SNRPA1 has been reported as the prognostic biomarker for diverse types of cancers, but still lack the report in ccRCC.

In the current study, we firstly confirmed the elevated level of SNRPA1 in both RNA-seq datasets and the clinical samples from our own institute. Among all the cancer types, SNRPA1 expression is correlated with the prognosis of ccRCC patients in OS, PFI, DSS, and DFI. After the univariate and multivariate Cox regression among SNRPA1 and clinical features, we revealed that SNRPA1 is the independent prognostic factor for ccRCC, no matter in which clinical subtype. We also conducted the knockdown studies of SNRPA1 in two ccRCC cell lines, 786-O and OS-RC-2. We revealed that the knockdown of SNRPA1 can inhibit the cell proliferation, migration, and invasion of ccRCC. Feng et al. (26) demonstrated that they revealed that SNRPA1 was highly expressed in HCC tissue compared with normal adjacent liver tissues, upregulation of SNRPA1 was correlated with the clinical stage of HCC and the overall survival of HCC patients, and knockdown of SNPRA1 inhibited the cell proliferation, colony formation, and xenografted tumorigenesis, which is consistent with our findings in ccRCC. Yuan et al. (16) also observed the increased value of SNPRA1 in prostate cancer patients; SNRPA1 inhibition also decreased tumor cell migration and colony formation.

Further mechanism study let us know that SNRPA1 impacted the tumorigenesis of ccRCC through RNA splicing, DNA replication, and activation of ATPase and methyltransferase, as well as the activation of E2F targets and MYC targets, p53 signaling, and epithelial–mesenchymal transition (EMT) differentiation. Chen et al. (27) reported a new lncRNA RMRP, which could interact with SNRPA1 and sequester it in the nucleus, and then nuclear SNRPA1 interacts with p53 and enhances MDM2-induced proteasomal degradation of p53, finally resulting in the promotion of tumorigenesis in colorectal cancer. ccRCC is characterized by the frequent silenced VHL gene and activated HIF-VEGF pathway, finally leading to the advanced metastasis and invasion with the vascularization within the microenvironment. Non-specific receptor tyrosine kinase inhibitors (RTKIs) are the first-line therapy for patients with progressive and metastatic renal cancer, such as sorafenib and sunitinib (28). Recently, several studies also reported the potential mechanisms of the increased resistance to RTKI treatment. Hwang et al. (29) reported that the cell cycle and EMT were significantly upregulated in the treated tumor samples compared with the pretreatment samples. Bao et al. (30) reported that angiopoietin-like protein 3 bound to focal adhesion kinase can attenuate the ubiquitination of p53, which contributed to cellular apoptosis and enhanced sorafenib response. Peng et al. (31) also reported the activation of EMT course and AKT/GSK-3β signaling pathway in sunitinib-resistance ccRCC. In the current study, we observed the activation of EMT and p53 pathways in high SNRPA1 patients and also revealed that patients with the lower level of SNRPA1 are more suitable by sunitinib treatment; results from the *in vitro* study also revealed that knockdown SNRPA1 can enhance the cell-killing effects of sunitinib. Therefore, the inhibitor of SNRPA1 is the promising new drug for the clinical treatment of sunitinib resistance patients.

ccRCC is reported to be a malignancy with high immunogenicity, which has been proven infiltrated by a large amount of immunocytes, including macrophage, NK cells, and T cells (32, 33). Especially, the disrupting activation of antigen presentation and the ruin of the immune system were impacted by the abnormal transformation of dendritic cells and the inactivation of T cells (34, 35). Several studies illustrated that the abundance of tumor-infiltrating lymphocytes and CD8+ T cells is inversely correlated with the prognosis of ccRCC patients (36–38). As for the immunocyte infiltration and immunotherapy, several studies already reported the application of anti-PD-1 or anti-PD-L1 therapy in ccRCC patients. Nivolumab is regarded as the standard of treatment strategy for advanced ccRCC and widely used in clinical trials (39). Furthermore, it is reported that the expressions of CTLA-4, PD-1, LAG-3, PD-L1, PD-L2, IDO1, and IL-10 were correlated with immunosuppression of the tumor microenvironment (40–42). Liu et al. (43) reported that CTLA4 was upregulated in ccRCC tissues and closely related to the disease progression as well as a poor prognosis, and high CTLA4 increased the infiltration of CD8+ T cells and Tregs, which resulted in an immunosuppressed phenotype of the immune microenvironment. Braun et al. (44) reported that exhausted CD8+ T cells and M2-like macrophages co-occurred in advanced disease and expressed ligands and receptors that support T cell dysfunction and M2-like polarization; the immune dysfunction circuit resulted with a worse prognosis. In the current study, we revealed the positive association between SNRPA1 and immunocyte infiltration, as well as the immune checkpoints, especially PD-1, PD-L1, and CTLA4. Along with the literatures mentioned above, a high level of immune checkpoints resulted in the immune dysfunction, and patients with a lower level of SNRPA1 respond more from anti-PD-1 immunotherapy. Therefore, the combination blockade of SNRPA1 with immunotherapies might obtain synergistic antitumor activity.

## Conclusion

We recognized the prognostic value of SNRPA1 in ccRCC by bioinformatic analyses and *in vitro* experiments. Elevated SNRPA1 was presented in ccRCC and acted as the independent risky prognostic factor. Combination blockage of SNRPA1 can provide the synergistic antitumor effect to both sunitinib treatment and anti-PD-1 immunotherapy.

## Data Availability Statement

The original contributions presented in the study are included in the article/**Supplementary Material**. Further inquiries can be directed to the corresponding authors.

## Author Contributions

AJ, JM, WG, and ZZ have contributed equally to this work. BL, LQ, and LW conceptualized and designed this study. XG, JW, and ZW wrote the first draft of the manuscript. All authors contributed to the article and approved the submitted version.

## Funding

This study was funded by the National Natural Science Foundation of China (82072812 to LW; nos. 81772740 and 82173345 to LQ) and the Foundation for Distinguished Youths of Jiangsu Province (no. BK20200006 to LQ).

## Conflict of Interest

The authors declare that the research was conducted in the absence of any commercial or financial relationships that could be construed as a potential conflict of interest.

## Publisher’s Note

All claims expressed in this article are solely those of the authors and do not necessarily represent those of their affiliated organizations, or those of the publisher, the editors and the reviewers. Any product that may be evaluated in this article, or claim that may be made by its manufacturer, is not guaranteed or endorsed by the publisher.

## Supplementary Material

Supplementary Table 1 Clinical features of the patients enrolled in SMMU cohort.

Supplementary Figure 1 Association between SNRPA1 expression and tumor stage in pan-cancer.

Supplementary Figure 2 Prognosis value of SNRPA1 in out-house datasets.

Supplementary Figure 3 SNRPA1 positively linked with the increased score of immune exclusion.

Supplementary Figure 4
**(A)** Know-out effect of SNRPA1 in 786-O and OS-RC-2 cell lines. **(B)** Cell proliferation of 786-O and OS-RC-2 after transfected with NC and SNRPA1 cas9-sgRNA plasmid, ***P<0.001.

Supplementary Figure 5
**(A)** Correlations of SNRPA1 expression with HALLMARKS enrichment score in 33 cancer types. **(B-G)** Correlation between SNRPA1 expression and HALLMARK_MYCC_TARTGETS_V2, HALLMARK_MYCC_TARTGETS_V1, HALLMARK_MTORC1_SIGNALING, HALLMARK_G2M_CHECKPOINT, HALLMARK_E2F_TARGETS and HALLMARK_DNA_REPAIR pathway in pan-cancer.
