# Supplementary figures and images for: Elevated SNRPA1, as a Promising Predictor Reflecting Severe Clinical Outcome via Effecting Tumor Immunity for ccRCC, Is Related to Cell Invasion, Metastasis, and Sunitinib Sensitivity
Source: Front Immunol. 2022 Feb 23;13:842069. doi: 10.3389/fimmu.2022.842069 (PMC8904888; doi:10.3389/fimmu.2022.842069)

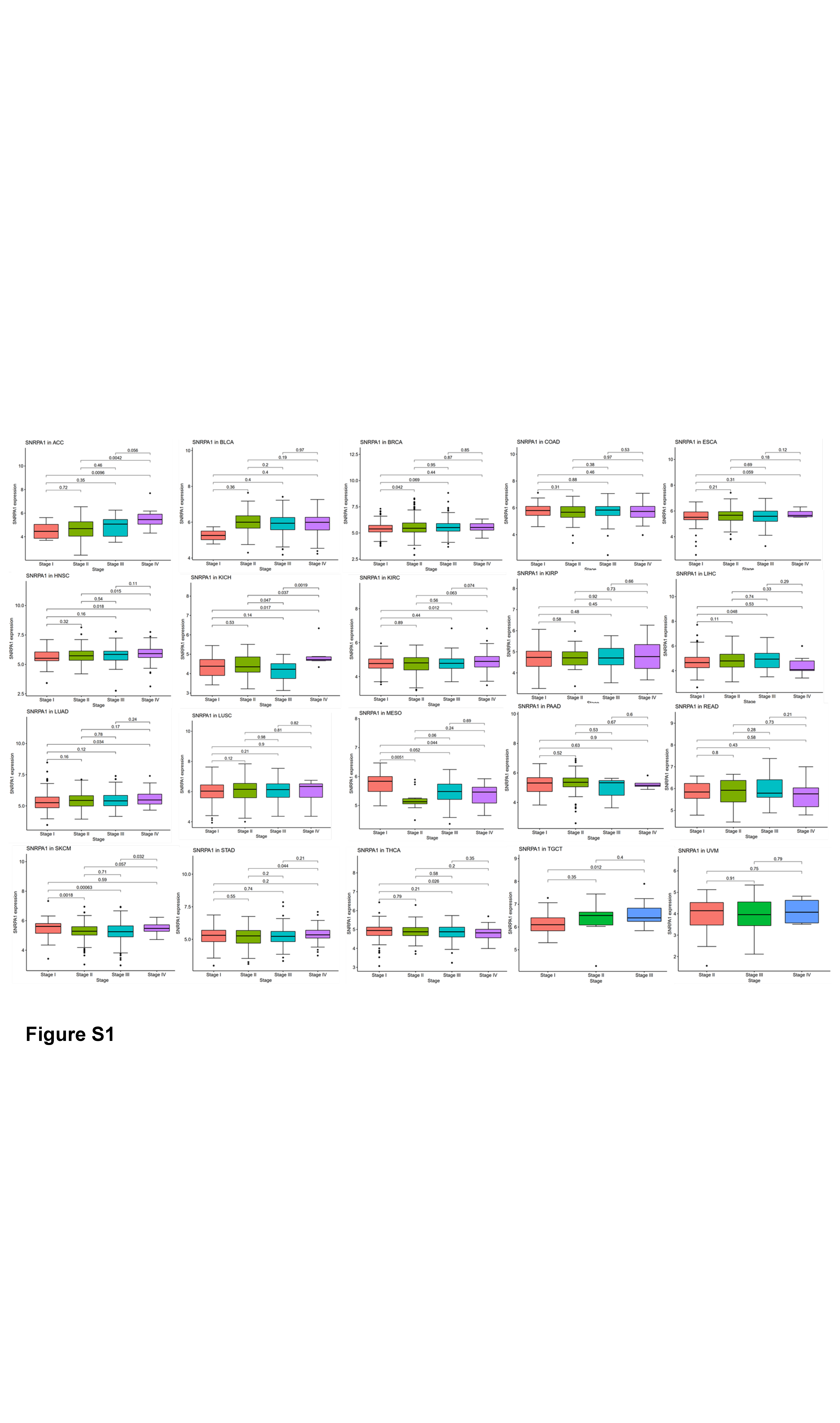

Supplement: Supplementary Figure 1 — Association between SNRPA1 expression and tumor stage in pan-cancer. [file Image_1.tiff]

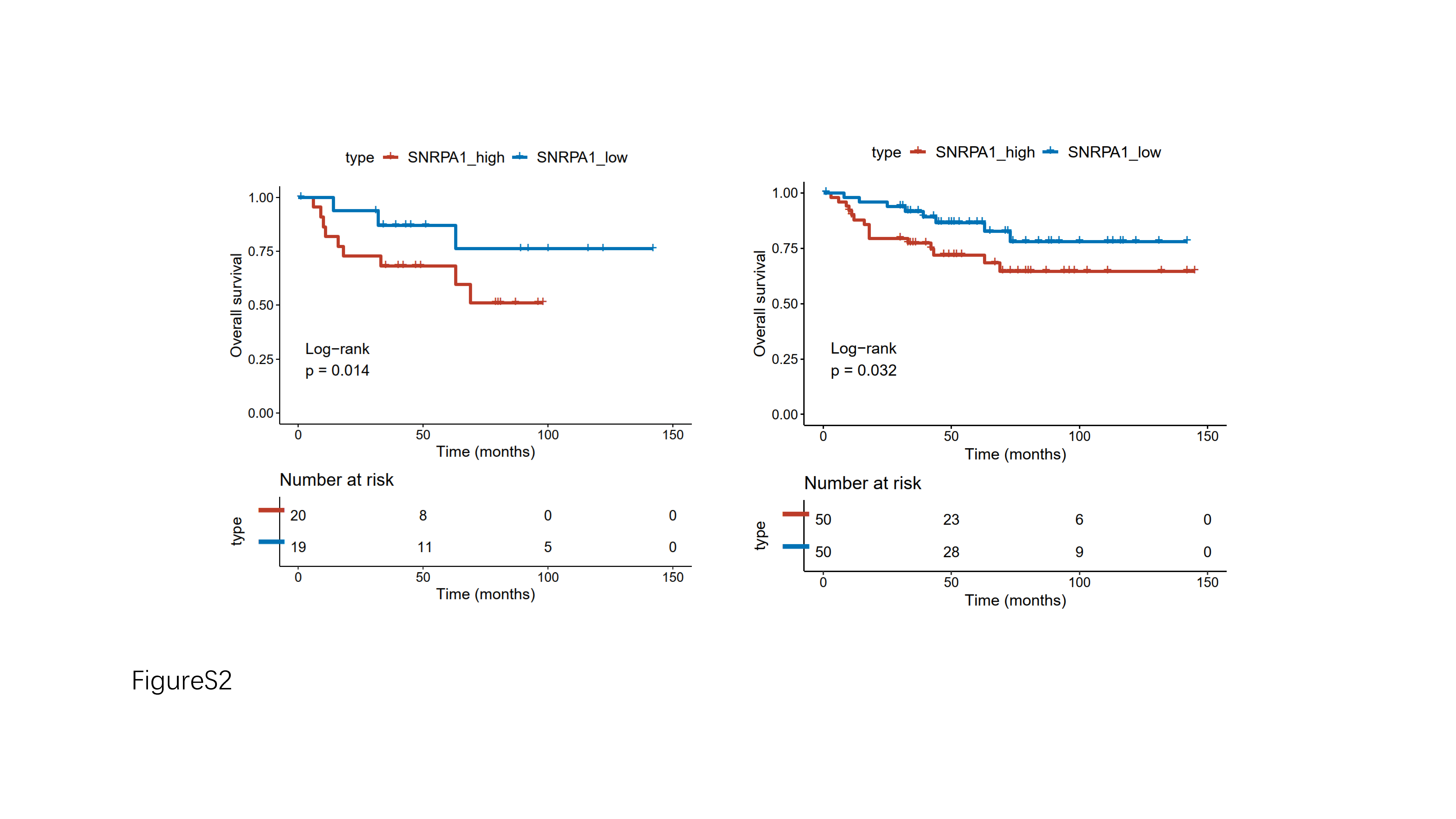

Supplement: Supplementary Figure 2 — Prognosis value of SNRPA1 in out-house datasets. [file Image_2.tif]

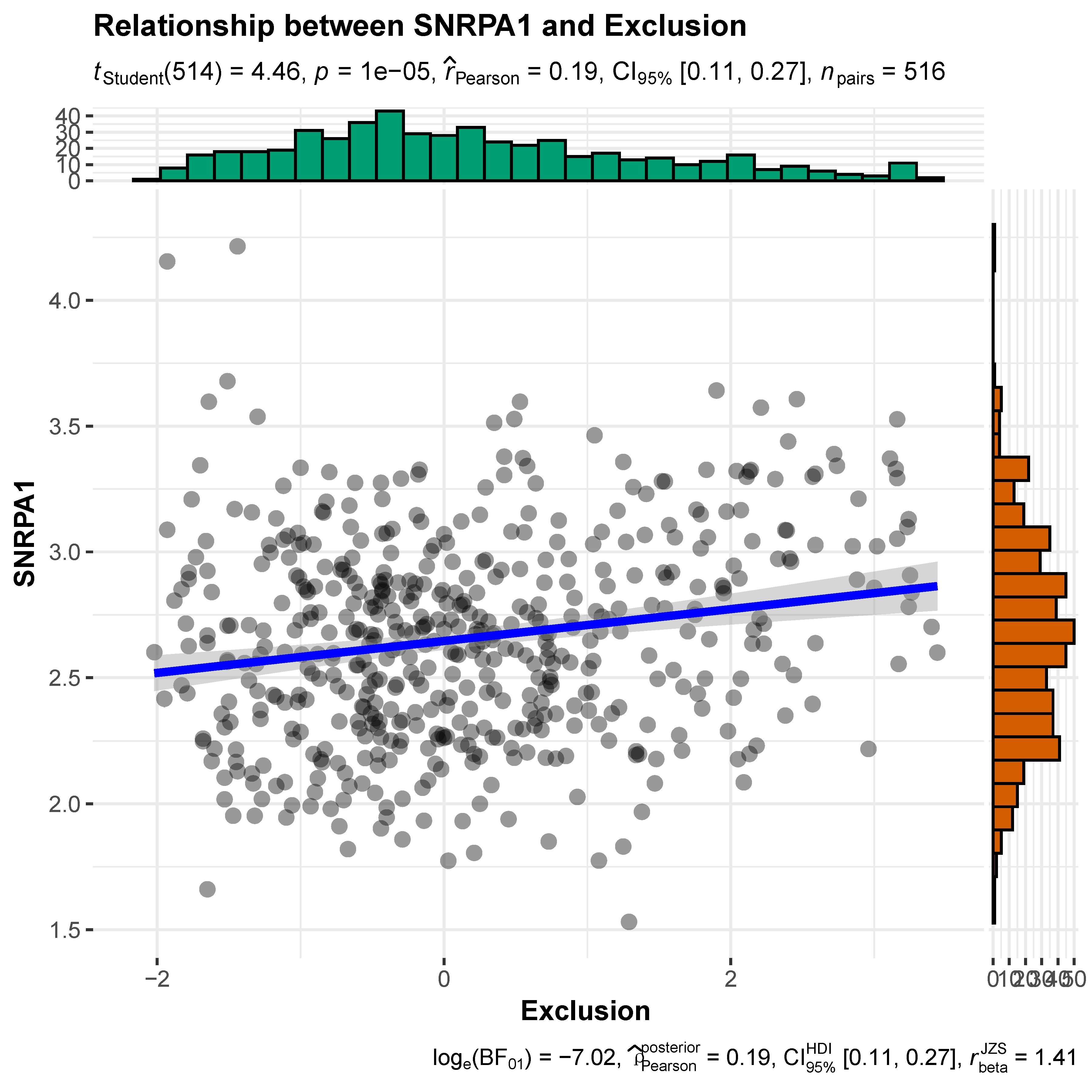

Supplement: Supplementary Figure 3 — SNRPA1 positively linked with the increased score of immune exclusion. [file Image_3.tif]

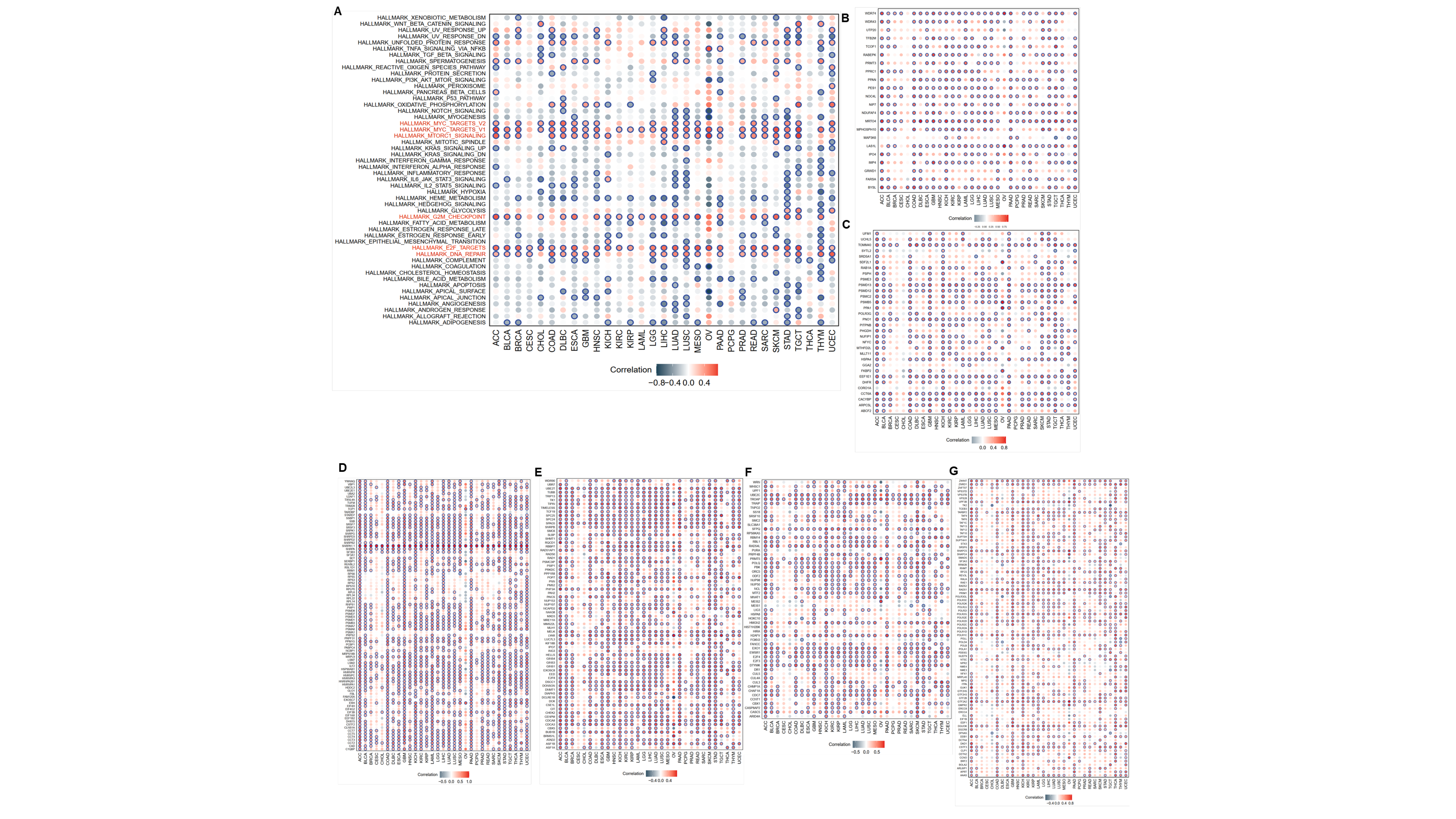

Supplement: Supplementary Figure 5 — (A) Correlations of SNRPA1 expression with HALLMARKS enrichment score in 33 cancer types. (B-G) Correlation between SNRPA1 expression and HALLMARK_MYCC_TARTGETS_V2, HALLMARK_MYCC_TARTGETS_V1, HALLMARK_MTORC1_SIGNALING, HALLMARK_G2M_CHECKPOINT, HALLMARK_E2F_TARGETS and HALLMARK_DNA_REPAIR pathway in pan-cancer. [file Image_5.tif]
